# Proteolytically Derived Endogenous Angioinhibitors Originating from the Extracellular Matrix

**DOI:** 10.3390/ph4121551

**Published:** 2011-12-02

**Authors:** Chandra Shekhar Boosani, Yakkanti A. Sudhakar

**Affiliations:** 1 Cell Signaling, Retinal and Tumor Angiogenesis Laboratory, Department of Genetics, Boys Town National Research Hospital, Omaha, NE 68131, USA; E-Mail: ChandraShekhar.Boosani@boystown.org; 2 Department of Biochemistry and Molecular Biology, University of Nebraska Medical Center, Omaha, NE 68198, USA

**Keywords:** extracelluar matrix, endogenous angioinhibitors, tumor angiogenesis, arresten, canstatin, tumstatin, endostatin, endorepellin, angiostatin, prothrombin kringle domain 2, thrombospondin, vasohibin, PEX domain, integrin signaling

## Abstract

Angiogenesis, a neovascularization process induced from the existing parent blood vessels, is a prerequisite for many physiological and pathological conditions. Under physiological conditions it is regulated by a balance between endogenous angioinhibitors and angioactivators, and an imbalance between them would lead to pathological conditions such as cancer, age-related macular degeneration (AMD), diabetic retinopathy, cardiovascular diseases, *etc.* Several proteolytically generated endogenous molecules have been identified which exhibit angioinhibition and/or antitumor activities. These angioinhibitors interact with endothelial and tumor cells by binding to distinct integrins and initiate many of their intracellular signaling mechanisms regulating the cell survival and or apoptotic pathways. The present review will focus on the extracellular matrix derived angioinhibitors, and their mechanisms of actions that point to the clinical significance and therapeutic implications.

## 1. Introduction

The term angiogenesis refers to the growth of new blood vessels from the pre-existing ones [1]. Angiogenesis is required for normal growth and body development that involves several physiological processes such as wound healing, tissue and organ regeneration, embryonic development, *etc.* This physiological angiogenesis is regulated with a tight control on endothelial cells and growth factors, however under pathological conditions such as cancer, AMD, diabetic retinopathy, cardiovascular diseases, *etc.*, an abnormal growth of new blood vessels occurs, which essentially contributes to the severity of the disease. In early 70s Professor Judah Folkman hypothesized that tumors require additional oxygen and nutrients for their rapid growth and thus induce growth of new blood vessels towards the growing tumors (tumor angiogenesis). Since under normal physiological conditions angiogenesis is tightly controlled, it was obvious that there exists endogenous angioinhibitors that play vital role in this tight control of physiological angiogenesis. Thus with the discovery of the thrombospondins (the first endogenous angiogenesis inhibitor to be identified) a new branch of basic research has emerged and at present about 27 endogenous antiangiogenic molecules have been identified, of which, many of them are in preclinical trials. Antiangiogenic therapy is now gaining its significance as the fourth treatment modality for cancer besides surgery, chemotherapy and radiotherapy. Several inhibitors and monoclonal antibodies were developed to prevent tumor angiogenesis and are currently in different phase trials proving their efficacy in cancer treatment. However, tumors may counterbalance the inhibitory effects of angioinhibitors by secreting increased amounts of angiogenic factors [2-4]. Thus to overcome such inhibition endogenous angioinhibitors appear more promising as they would not invoke any defense mechanism. However in cancer cells when thrombospondin-1, endostatin, and tumstatin are over expressed, tumor cells were found to escape angiogenesis inhibition by up-regulation of various proangiogenic factors [5]. Yet with the increasing list of endogenous antiangiogenic molecules each with a unique mechanism of action and with varied potential of inhibiting de novo angiogenesis in different pathological conditions, the chances of preventing tumor angiogenesis are high, from this perspective the present review discusses on the endogenous angioinhibitors that are derived from the extracellular matrix as a result of the proteolytic activity of several endoproteases and details the possible integrin receptors and their mechanism of actions.

With the exception of PEX domain and vasohibins, the below described angioinhibitors could be classified as either extracellular matrix derived (such as arresten, canstatin: tumstatin: endostatin: endorepellin) or plasma derived molecules (that include: angiostatin: prothrombin kringle domain-2: thrombospondins).

## 2. Extracellular Matrix Derived Angioinhibitors

### 2.1. Arresten (α1(IV)NC1)

Arresten was isolated as a 26-kDa endogenous molecule from the C-terminal noncollagenous (NC1) domain of the α1 chain of type IV collagen [6]. It was shown to inhibit bFGF-induced, endothelial cell proliferation and tube formation besides reducing tumor metastases and human xenograft tumors in arresten treated nude mice. They also showed decreased endothelial cell binding to arresten coated plates when cells were treated with α1 and β1 integrin antibodies, suggesting that α1β1 is a possible integrin receptor for arresten. Bacculovirus-expressed recombinant arresten was found to inhibit tube formation, proliferation and migration of HUVECs in an α1β1 integrin dependent manner, also pretreatment of arresten to HUVECs inhibited FAK/c-Raf/MEK/ERK1/2/p38 MAPK pathway [7]. The authors also showed that arresten, when treated to endothelial cells cultured on type IV collagen inhibited hypoxia induced expression of HIFα and VEGF by inhibiting ERK1/2 and p38 MAPK activation. Their study also reports that SCC-PSA1 tumors had decreased number of CD31 positive vasculature when treated with recombinant arresten, indicating that arresten inhibits tumor angiogenesis *in vivo*. Arresten was also shown to induce apoptosis by decreasing the expression of Bcl-2 and Bcl-_xL_ in endothelial and tumor cells in mice [8]. More interestingly the active site of arresten was identified through deletion mutagenesis to be localized within the C-terminal subunit. Endothelial cell proliferation induced by bFGF was found to be significantly inhibited by arresten in a dose and time dependent manner [9]. Increased secretion and activation of MMP-2 by bFGF was also shown to be inhibited when endothelial cells were treated with arresten in a dose-dependent manner, however the levels of MMP-2 mRNA were not affected, indicating that arresten interacts with MMP-2 and inhibits its activation. The mechanism shown here was that arresten covalently binds to proMMP-2 and inhibits its auto activation. Arresten was also reported to promote apoptosis by caspase-3/PARP activation and by negatively regulating FAK, p38-MAPK phosphorylation, Bcl-2, and Bcl-_xL_ expression in mouse retinal endothelial cells (MREC) [10]. Interestingly they also found that arresten inhibited VEGF-induced MREC migration and proliferation, but not MRPEC proliferation.

### 2.2. Canstatin (α2(IV)NC1)

Canstatin was isolated as a 24-kDa fragment from the C-terminal noncollagenous domain of the α2 chain of type IV collagen, which significantly inhibited endothelial cell migration and tube formation without affecting nonendothelial cells, besides inducing apoptosis and suppressing *in vivo* human xenograft prostate adenocarcinoma tumors in nude mice [11]. The apoptotic activity of canstatin was shown to be mediated by binding to αVβ3 and αVβ5 integrins which initiate cell death via activation of procaspase 8 and 9 which in turn lead to activation of caspase-3 [11-13]. Treatment with canstatin increased expression of Fas ligand and decreased FLIP protein binding to FADD and caspase-8, inducing death receptor mediated apoptosis [11,13,14]. Canstatin localizes on the MDA-MB-231 tumor cells and increases mitochondrial caspase-9 activity, thereby inducing apoptosis [12]. Through immunoprecipitation studies using antibodies against αVβ3 and αVβ5 it was shown that canstatin binds to both these integrins on the endothelial surface, and has a higher antiangiogenic potential than angiostatin [12]. When endothelial cells were treated with canstatin, phosphorylation of FAK, Akt, and downstream targets such as mTOR, 4E-BP1, and p70s6k were found to be inhibited, indicating the caspase-9 mediated apoptotic activity of canstatin [13]. The amino acids 1–89 of canstatin was shown to be more potent that canstatin itself and this region was found to specifically inhibit endothelial cell proliferation and induced apoptosis, besides suppressing growth of B16 murine melanoma tumors [15]. The same group also showed that the C-terminal 157–227 amino acid region of canstatin inhibits endothelial cell proliferation and apoptosis, but the apoptosis-inducing activity was much lower than the 1–89 amino acid region of canstatin with similar tumor suppression activity [16]. In another interesting study which is a first report of its kind, the ^131^I radiotherapy was combined with angiogenesis inhibition, using both sodium iodide symporter (NIS) and canstatin that was delivered by adenovirus. This dual therapy was found to strongly impede the growth of xenograft and spontaneous tumors in mice [17]. The recombinant canstatin not only was shown to inhibit tube formation in HUVECs and lymphatic endothelial cells, but also reduced the growth of oral squamous cell carcinoma tumors in mice models [18]. Using the novel oncolytic conditionally-replicating adenovirus (CRAd) in which the E1B-55kDa gene for selective replication in tumor cells was replaced with canstatin, the synergistic effects of oncolytic therapy and anti-angiogenesis therapy for pancreatic cancer was also reported [19]. By combining tumor necrosis factor-related apoptosis-inducing ligand (TRAIL) gene therapy and canstatin, inhibition of human breast tumors in nude mice was observed [20]. Recently, the same group has identified that recombinant canstatin inhibits angiopoietin-1-induced angiogenesis and lymphangiogenesis [21]. In their study they also identified that expression of angiopoietin-1 in CT-26 cells under hypoxic conditions is inhibited by canstatin and affects both angiogenic and lymphangiogenic signaling induced by angiopoietin-1, which is presumed to be mediated through integrin-dependent FAK signaling induced by angiopoietin-1/Tie-2 and/or VEGFR-3. They also showed the antiangiogenic effects of canstatin in inhibiting alkali burn-induced corneal neovascularization in mice [21].

### 2.3. Tumstatin (α3(IV)NC1)

Tumstatin was isolated as a 28-kDa noncollagenous NC1 domain that was proteolytically cleaved from the C-terminal region of α3 chain of type IV collagen [22]. The region between 185–203 amino acids of tumstatin was found to inhibit activation of human polymorphonuclear monocytes [23]. Also the region between 54–132-amino acids corresponding to Tum-5 peptide was shown to inhibit tube formation and induce cell cycle arrest at G1 phase in endothelial cells, besides inhibiting human prostate cancer growth and angiogenesis in nude mice [24]. Tumstatin was reported to inhibit bFGF-induced proliferation of HUVECs, and melanoma cells, besides inducing apoptosis in endothelial cells and inhibiting neovascularization in matrigel plugs and *in vivo* tumor growth in different murine cancer types [22,24-27]. The antiangiogenic properties of tumstatin have been reported through several different pathways. Tumstatin binds to αVβ3 integrins through an RGD-independent mechanism and inhibits CAP-dependent protein translation by FAK/PI3K/Akt pathway down regulating mTOR, 4E-BP1, and eIF-4E [26]. This specific activity of Tumstatin was found in the region between 69–98 amino acids. The same integrins were also reported to be involved in regulating the antiangiogenic functions through PTEN/Akt pathway [28]. Deletion of tumstatin and thrombospondin-1 in mice lacking the p53 tumor suppressor gene showed increased incidence and reduced latency of angiogenic lymphomas [29]. Also intratumoral expression of Tum1 showed significant repression of the growth of Huh-7 (hepatocellular carcinoma) tumors in nude mice with decreased CD34 positive vessels indicating the antiangiogenic potential of Tum1 that could be used in gene therapy [30]. A fusion protein comprising the 88 amino acid sequence from tumstatin 45–132 with TNFα showed inhibition of angiogenesis and tumor-cell viability *in vitro*, also intratumoral injection of this Tumstatin45-132-TNFα protein showed decreased blood-vessel density in xenograft F6 tumors in mice [31]. In oral squamous cell carcinoma animal model, the effects of tumstatin in inhibiting tumor growth was shown *in vivo*, although the tumors did not show total remission, the authors found decreased tumor microvessel density indicating that tumstatin delays the tumor growth and metastasis of oral squamous cell carcinomas [32]. The antiangiogenic properties of tumstatin were also shown to be mediated by its binding to integrins αVβ3 and α3β1, and regulation of the PI3-K/4E-BP1 pathway [33,34]. The expression of the proinflamatory molecule COX-2 was reported to be inhibited in integrin β3-null MLECs, and not in α3-null MLECs when treated with tumstatin, indicating that integrin α3β1 is a functional receptor for, umstatin in inhibiting hypoxic COX2 expression which is also a proangiogenic factor [35]. A ,umstatin peptide was shown to bind to αVβ3 integrins on proliferating endothelial cells in tumor endothelium, and in combination with anti-VEGF antibody (bevacizumab) it was shown to suppress renal cell carcinoma tumors [36]. The YSNSG cyclopeptide derived from umstatin showed inhibition of endothelial cell migration *in vitro* without affecting cell proliferation, this inhibition of cellular migration was reported to be mediated by a decrease in active MT1-MMP, u-PA and u-PAR expression [37]. Expression of Tumstatin45-132-TNFα showed inhibition of cellular proliferation and induction of apoptosis in prostate cancer cells in xenograft tumors [38,39].

### 2.4. Hexastatin (α6(IV)NC1)

Another noncollagenous domain derived from the sixth chain of type IV Collagen was identified as antiangiogenic, is about 228 amino acids in length and was found to inhibit angiogenesis and tumor growth affecting endothelial cell adhesion and migration which was mediated by αV and β1 integrins [33]. Hexastatin was also shown to inhibit proliferation of HUVECs and neovascularization in matrigel plugs besides inhibiting the growth of LLC and pancreatic tumors in mice [40]. However a detailed mechanistic study in identifying the signaling mechanisms involved in its antiangiogenic functions is yet to be identified.

### 2.5. Endostatin

Endostatin was discovered as a 20 kDa protein derived from the C-terminal fragment of type XVIII collagen which inhibited endothelial cell proliferation, angiogenesis and tumor growth [41]. The authors also identified the antitumor potential of endostatin in inhibiting growth of several tumor types such as Lewis lung carcinomas, T241 fibrosarcomas, B16F10 melanomas and hemangioendothelioma. Recently, tumor growth in many cancer types was found to be significantly reduced upon treatment with endostatin [42]. Lack of endostatin in humans results in Knobloch syndrome, an autosomal recessive disorder that results in blindness at birth due to failure of retinal development [43]. A similar condition was also reported in mice deficient in endostatin that fail to develop a vascularized retina [44]. The circulating levels of endostatin in healthy individuals was reported to be between 10 to 50 ng/mL which is equivalent to 0.5–2.5 nM however, elevated levels of endostatin were reported in several cancer types that include osteosarcoma, NSCLC, hepatocellular carcinoma, ovarian cancer, bladder cancer, head and neck squamous cell carcinoma, renal cell carcinoma, soft tissue sarcoma, acute myeloid leukemia and colorectal cancer. Such higher levels of endostatin in cancer patients was implicated as a prognostic factor indicating that endostatin may be used a therapeutic target for the treatment of these cancer types [45-56]. Murine hemangioendothelioma tumor cells (EOMA) from which endostatin was originally isolated secrete MMPs and procathepsin that gets activated to cathepsin in acidic medium and generates endostatin from type XVIII collagen which then exerts its antiangiogenic activity [57]. One of the many mechanisms by which endostatin regulates its anitangiogenic activity was thorough inhibition of matrix metalloproteinases2 (MMP-2)-mediated endothelial cell invasion, where endostatin binds to the catalytic domain of proMMP2 forming a stable complex [58-60]. Endostatin inhibits endothelial cell survival and migration by inhibiting VEGF121, VEGF165 and their cognate receptor KDR/FLK-1 [61,62]. Endostatin was also shown to inhibit PDGF mediated recruitment of perivascular cells affecting maturation of new blood vessels [63]. The many other mechanisms which enhance the antiangiogenic properties of endostatin include, blocking VEGFR2 signaling, suppressing Wnt signaling, catenin destabilization, or altering catenin/VE cadherin interactions in interendothelial cell junctions. A comprehensive signaling mechanism of endostatin involving Id/AP-1, HIF-1α, ephrin and TNF-α, NF-κB, STAT and Ets in cell proliferation, migration, survival, tube formation and apoptosis, besides coagulation cascades and adhesion molecule pathways was also reported [64]. Nucleolin on the cell surface to which endostatin binds with high affinity, was identified to be a vital receptor for endostatin to mediate its antiangiogenic and antitumor functions [65]. Nucleolin is a ubiquitous multifunctional protein critically involved in cell proliferation, chromatin organization, packaging of pre-RNA, rDNA transcription, and ribosome assembly [66-68]. Nucleolin is mobilized from nucleus to endothelial cell surface and is expressed only in new angiogenic blood vessels which is modulated by VEGF and ECM proteins [69]. Other mechanism by which endostatin functions were reported by inducing endothelial cell apoptosis, down regulating Bcl-2, and up regulating caspase-3 expression *in vitro* [70,71]. Endostatin binds to the endothelial cell surface integrins α5 and αV and prevents integrin dependent cell migration [72,73]. More precisely the integrin α5β1 was identified as a potential receptor for endostatin by which it regulates its outside in signaling [34]. Also the tumor suppressor functions of endostatin in knockout mice were reported where tumors grew 2- to 3-fold faster [74].

### 2.6. Endorepellin

Endorepellin was identified from the C-terminal functional region of Perlecan with the potential to inhibit angiogenesis, and was found to inhibit endothelial cell migration, tube morphogenesis and adhesion *in vitro*, besides inhibiting angiogenesis in matrigel plug and CAM assay *in vivo*. Endorepellin also affects cell adhesion in several cancer cells such as those derived from colon, neuroectoderm and mesenchyme [75]. The authors also showed that endorepellin is comprised of three LG modules and LG2 binds to endostatin, but this binding does not affect the antiangiogenic activity of either endostatin or endorepellin. Enzymes belonging to the BMP-1/tolloid-like proteinase family were reported to cleave endorepellin and liberate LG3 module, which contributes the antiangiogenic functions of endorepellin [76]. Endorepellin when administered intraperitoneally was reported to target the tumor vasculature in squamous cell carcinoma xenografts and in syngeneic LLC tumors, inhibiting tumor growth. A possible mechanism of caspase-3 activation during apoptosis initiating the release of cathepsin-L was identified to be vital for endorepellin proteolysis and LG3 production [77]. Endorepellin belongs to the RGD-independent and cation-independent class of molecules that bind to α2β1 integrin receptors.

**Figure 1:**
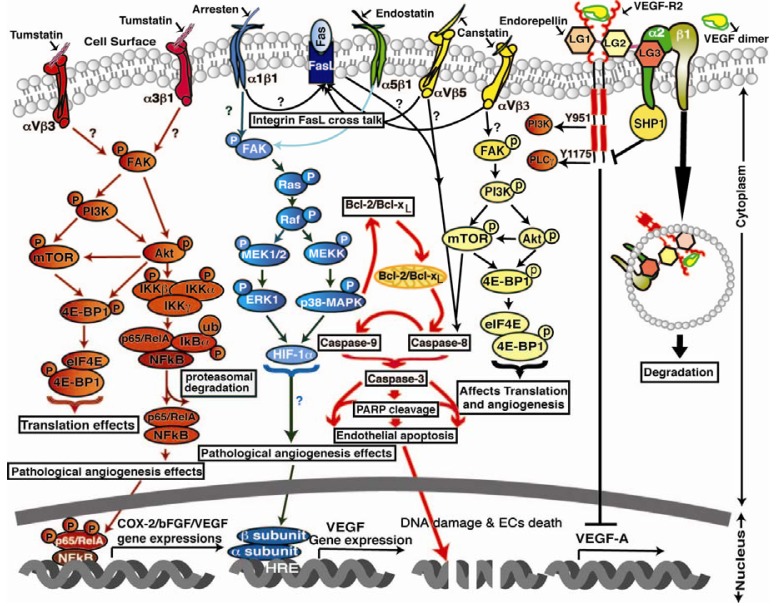
Illustration of extracellular matrix derived endogenous angioinhibitors.

Knockdown of integrin α2 subunit using siRNA significantly affected migration of endothelial and fibrosarcoma cells across collagen. Using a LLC xenograft model, it was also confirmed that the antitumorangiogenic activity of endorepellin is mediated through α2β1 integrins [78]. The angiostatic activity of endorepellin was reported to be greatly confined within the LG3 module which binds to α2-I integrin domain, a major binding site for collagen I [79]. It was concluded that interactions of endorepellin with α2β1 integrins causes increase in cAMP and activation of PKA and FAK, but not Erk1/Erk2, and leads to transient phosphorylation of p38 MAPK and Hsp27. Blockade of α2β1 integrins in fibroblasts was shown to inhibit the antiapoptotic response initiated by recombinant LG3 [80]. Endorepellin supports α2β1 integrin-mediated and Src-kinase-dependent platelet adhesion, but does not contribute to activation or platelet aggregation [81]. Endorepellin was shown to cause a rapid activation of the tyrosine phosphatase Src homology-2 protein phosphatase-1 (SHP-1), and provokes global dephosphorylation of several RTKs that are dependent on the presence of the integrin α2β1 [82]. TIMP-2 was reported to induce a substantial increase in cAMP levels with the activation of cAMP-dependent PKA in microvascular endothelial cells, fibroblasts, as well as in several transformed cells. The ability of endorepellin to activate SHP-1 with TIMP-2 was shown to induce a signaling cascade of events involving key angiogenic regulators such as RTKs, VEGFR2 and FGFR1 [83-86]. Recently, similar to TIMP-2, which binds to the α3β1 integrin and induces SHP-1, and in turn dephosphorylates several receptor tyrosine kinases, including VEGFR2 and FGFR1, endorepellin was also shown to activate the phosphatase SHP-1 but its antiangiogenic signaling was found to be mediated through α2β1 integrins unlike TIMP-2 which is mediated by α3β1 [87]. Also, endorepellin treated tumors were shown to be hypoxic with decreased metabolism, and decreased cell proliferation without inducing apoptosis or inhibiting wound healing [88]. The LG3 module of endorepellin was reported as a serological biomarker for breast cancer since its plasma levels were found low in breast cancer patients [89]. Signaling mechanisms of the extracellular matrix derived angioinhibitors are shown in Figure 1.

## 3. Plasma Derived Endogenous Angioinhibitors

### 3.1. Angiostatin

Angiostatin was identified as a 38 kDa fragment from the elastase digest of plasminogen which was isolated from the urine of LLC tumor bearing mice, with an half-life of 2.5 days [90]. Although with little ambiguity, angiostatin refers to the proteolytic fragment of plasminogen comprising five kringle domains, and depending on the protease used its molecular weight ranges between 38–55 kDa. Among the five kringle domains, kringle domain-4 was ineffective, unlike the other four domains that showed strong antiangiogenic functions in endothelial cells. A number of antiangiogenic properties of angiostatin have been identified, in endothelial cells angiostatin was shown to inhibit cellular proliferation, migration and tube formation on matrigel matrix. However, angiostatin was reported to have no effect on a variety of normal, neoplastic and nonendothelial cell lines such as 3T3 fibroblasts, bovine aorta smooth muscle cells, bovine retinal pigment epithelial cells, human fetal fibroblasts, and LLC carcinoma cells [91,92]. Angiostatin was shown to induce endothelial cell apoptosis *in vitro* by RGD independent activation of FAK besides inhibiting VEGF and bFGF mediated cellular migration and tube formation [93]. However the signal transduction mechanism of angiostatin upon treatment to microvascular endothelial cells, resulted in decreased activation of ERK 1 & 2 MAP kinases that were activated by VEGF and bFGF [94]. Intracranial administration of angiostatin was also shown to result in suppression of brain tumor growth and decreased tumor angiogenesis [95]. Mice lacking plasminogen, which is a precursor for angiostatin, showed spontaneous fibrin deposits with reduced fertility and survival indicating that plasminogen or plasmin are not essential for embryonic development [96]. The antiangiogenic functions of angiostatin were reported to be mediated by at least three different receptors that were identified on endothelial cell surface that include ATP synthase, angiomotin and integrin αVβ3, α4β1 and α9β1 [97-99]. The anticancer functions of angiostatin have been studied in several cancer types such as lung cancer, brain cancer, colon cancer, breast cancer, *etc.* With successful completion of PhaseI/II clinical trials of angiostatin for patients with progressive metastatic cancer and non-small-cell lung cancer, the results from phase III clinical trials of angiostatin are awaited in anticipation that the study would be completed by June 2012 as scheduled.

### 3.2. Prothrombin Kringle-2

The group led by Soung Soo Kim, have made vital studies in identification of prothrombin kringle-2 as an endogenous antiangiogenic molecule and made significant contributions to this area of research. Human prothrombin was digested with Factor Xa overnight and prothrombin fragments 1 and 2 are isolated as 30 kDa and 19 kDa proteins. The authors also identified that both the fragments inhibited bFGF induced endothelial cell growth in a dose dependent manner, and also inhibited *in vivo* angiogenesis through CAM assay [100]. Earlier the same group has reported the antiangiogenic functions of prothrombin kringle-2 domain from rabbits, which is a first report on its discovery [101]. Recombinant prothrombin kringle domains with antitumor properties, were studied using LLC tumor cells and reported that treatment with *E. Coli* expressed recombinant prothrombin kringle domain-2 not only inhibited tumor growth significantly but also prevented tumor metastasis [62]. The authors also detailed the mechanism by which prothrombin kringle domains are generated from prothrombin. Prothrombin is composed of 581 amino acids which when digested with Factor Xa results in two fragments 1–273 (amino terminal fragment) and 274–581 (active thrombin). Active thrombin then cleaves the amino terminal fragment and releases prothrombin kringle fragment-1 (1–155 amino acids) and prothrombin kringle fragment-2 (156–273 amino acids). Also the same group has identified two peptides NSA7 and NSA8 that were derived as C-terminal truncation products of NSA9 which was originally identified from prothrombin kringle-2 fragment. Although all the three peptides have significant antiangiogenic activity, NSA7 showed considerably higher effect than NSA8 and NSA9 as compared using cell proliferation inhibition assay, *in vivo* CAM angiogenesis assay, tube formation and migration of HUVEC cells. The peptide NSA7 was also found as an effective inhibitor for proliferation of B16F10, LLC and L929 tumor cells and gets internalized into endothelial and tumor cells more easily [102]. Endothelial cells when treated with prothrombin kringle-2 showed dose dependent inhibition of cellular migration and adhesion to ECM proteins especially using vitronectin matrix suggesting that αVβ3 could be a possible integrin receptor for prothrombim kringle-2 [103]. Mice treated with prothrombin kringle-2 showed resistance to melanoma pulmonary metastasis as they exhibited less metastatic colonies with small and isolated tumors, and also helped in restoring the acute lung injury associated with B16F10 melanoma metastasis to normal phenotype. Also, prothrombin kringle-2 was found to inhibit VEGF expression in type I and type II pneumocytes, endothelial cells and metastatic tumor cells with diminished CD31 expression which would have caused the inhibition of B16F10 melanoma metastasis associated with tumor neovascularization. Tumor cell derived MMP-2 or MMP-9 were reported to elicit secretion of soluble VEGF from the ECM [58,104]. Treatment with prothrombin kringle-2 decreased expression of MMP-2 and MMP-9 in the bronchiolar epithelial cells, pneumocytes, endothelial cells, and metastatic tumor cells of B16F10 melanoma, suggesting the possible mechanism of prothrombin kringle-2 antitumor actions [103]. Previously it was reported that activated microglia produces reactive oxygen species, resulting in oxidative damage and causes severe pathology in neurodegenerative diseases [105-108]. Prothrombin kringle-2 was shown to act as an endogenous microglial activator and exerts neurotoxicity in the cortex *in vivo*. Prothrombin kringle-2 induced up regulation of cytosolic protein p67phox co-localized within activated microglia in the cortex and activated microglial NADPH oxidase, enhanced reactive oxygen species production and protein oxidation which resulted in neurodegeneration in the cortex. However, prothrombin kringle-2 failed to cause neuronal loss in neuron enriched cortical cultures devoid of microglia suggesting that the activated microglia are required for prothrombin kringle-2 induced neurotoxicity. Supporting this observation, the authors also report that there is a concomitant increase in the level of nitrite formed from NO and TNF-α in cortical microglia cultures when treated with prothrombin kringle-2. Interestingly, the authors also identified that prothrombin kringle-2 treated cortex showed expression of iNOS and IL-1β *in vivo* [109,110].

### 3.3. Thrombospondins

**Figure 2:**
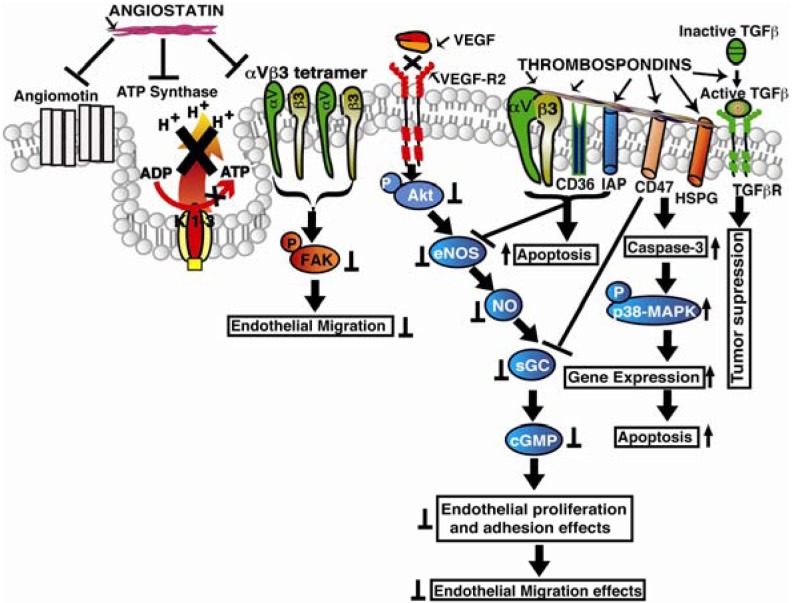
Illustration of non-collagenous extracellular matrix derived angioinhibitors.

Baenzinger in 1971 identified the presence of a high molecular weight thrombin sensitive protein when thrombin is added to intact platelets [111]. Later it was characterized as a high molecular weight glycoprotein isolated from human blood platelets and coined the term “thrombospondin” [112]. Thrombospondins are a family of extracellular matrix glycoproteins consisting of five members (TSP-1 to TSP-5) whose functions have been implicated in treating several cancer types. Among the five thrombospondins, TSP-1 and TSP-2 have equivalent domain structures and are widely studied. TSP-1 was reported as the first naturally occurring angiogenesis inhibitor to be identified for having angiostatic functions. The mechanisms by which TSPs exert their antiangiogenic functions include direct effects on inhibiting endothelial cell migration, apoptosis, or indirectly by inhibiting expression of growth factors, cytokines and proteases that regulate angiogenesis. TSP-1 and TSP-2 inhibit growth factors induced cell cycle progression by arresting the cells in the G0/G1 phase, and this inhibition is presumed to be independent of caspase activity. TSP-1 induces endothelial cell apoptosis by up regulating Bax and down regulating VEGF-mediated Bcl-2 expression [113]. CD47 (integrin-associated protein) was shown to impact angiogenesis to a large extent since binding of CD47 with TSP1 and other ligands inhibits VEGFR2 phosphorylation and angiogenesis. It was found that the C-terminal region of TSP-1 binds to CD47 and interacts with cell surface integrins [114]. TSP-1 was shown to bind to CD47 and regulates nitric oxide synthesis in both normal and pathological events [115]. Analysis of wound bed vascularity in TSP-1 and CD47 null mice showed increased angiogenesis indicating their essential role in antiangiogenesis [116]. Recently it was also reported that CD47 interacts with VEGFR2 receptor [117]. Thrombospondin type 1 repeats (TSRs) were found to be present in over 100 different proteins in the human genome, and the presence of these repeats has been correlated with the ability of these proteins to inhibit tumor angiogenesis and tumor growth [118,119]. The receptors for the TSR repeats in TSP-1 were identified as CD36, β1 integrins and TGF-β. The CD36 and TSP-1 interactions were reported to down regulate VEGF receptor-2 and p38 MAPK phosphorylation, inhibiting VEGF induced functions [120]. Also the interactions between TSRs and β1 integrins were shown to result in inhibition of endothelial cell migration [121]. The TSRs induced cell migration was also reported to be inhibited when treated with integrin α3, α5 and PI3 Kinase antagonists. The interaction between TSRs with CD36 was shown to result in endothelial cell apoptosis presumably mediated through Fyn and c-Jun N-terminal kinase (JNK) pathway [122,123]. The TSR sequence KRFKQDGGWSHWSPWSSC was reported to inhibit proliferation of both endothelial cells and tumor cells besides inhibiting angiogenesis in retinopathy and in solid tumors [124-126]. Also 18 functional peptides that were derived from type I thrombospondin repeat sequences were found to inhibit proliferation and migration of HUVECs when used up to 40 μg/mL concentrations. TSRs in TSP-1 were reported to bind to the type II fibronectin repeats of MMP-2 and inhibit its activation [127]. Also, in transgenic mice that over-express TSP-1 in the mammary glands, the levels of active MMP9 were found lowered in the developed tumors [128]. In TSP-2 null mice, implanted tumors showed increased tumor vascularization indicating its role in tumorangiogenesis [129]. Also overexpression of TSP-1 was shown to suppress tumor angiogenesis and metastasis but was reported to have no effect on lymphangiogenesis [130]. However, using CD36 null mice recently it was shown that TSP-1 inhibits lymphangiogenesis by binding to monocytes, and treatment of TSP-1 to macrophages resulted in suppression of lymphangiogenic factors VEGF-C and VEGF-D [131]. Signaling mechanisms of plasma derived endogenous angioinhibitors are shown in illustration in Figure 2.

## 4. Endogenous Angioinhibitors Identified from Other Sources

### 4.1. PEX Domain

MMPs are a family of zinc-dependent matrix-degrading enzymes with a wide variety of ECM proteins as substrates [132-134]. These MMPs were reported to have a prominent role in cellular invasion of several cell types, and an uncontrolled proteolysis of matrix by these MMPs would lead to severe pathological conditions [134]. However, a tight control was reported to exist *in vivo* that regulates this mechanism of matrix degradation during physiological angiogenesis. The noncatalytic C-terminal domain of MMP-2 (Hemopexin domain or PEX) was reported to interact with αVβ3 integrins on endothelial cell surface and prevents MMP-2 from binding to αVβ3 integrins which are key regulators of angiogenesis [135]. The authors also identified that PEX is a naturally occurring breakdown product of MMP-2 with detectable levels of PEX under normal physiological conditions and during retinal neovascularization and in *in vivo* tumors suggesting its vital role in angiogenesis and vasculogenesis, proving its antiangiogenic activity. It was also shown that αVβ3 integrins on CS-1 melanoma cells promotes collagenolytic activity that can be blocked using recombinant PEX domain, indicating that addition of PEX to αVβ3 expressing cells prevents binding of MMP-2 to the cell surface. Using lentiviral vectors, expression of PEX was shown to block bFGF induced MMP-2 activation, cell migration, proliferation, tube formation and CAM angiogenesis besides inhibiting human melanoma (M21L) tumor growth in nude mice [136]. The same group also showed that upon systemic administration of PEX, sustained inhibition of glioma tumors over a prolonged period of time, and the histological analysis showed decreased vascularity in tumors, with an increase in apoptosis [137]. The antitumor effects of PEX were evaluated by transfecting human neural stem cells and pTracer vector with PEX and reported inhibition of proliferation and migratory ability of PEX-producing cells *in vitro* [138]. It is interesting to see that neural stem cells were used here to deliver PEX into the xenograft tissue, and as reported, the transfected stem cells migrated to the tumor site and inhibited angiogenesis without inducing apoptosis or inhibiting cell motility. Recently, fusion of PEX domain with the N-terminal signal peptide of MMP-9 and stable transfection of SNB19 cells with the fusion construct showed secretion of PEX domain, and the cultured condition medium when treated to endothelial cells showed down regulation of MMP-9, VEGF and VEGFR2, induced cell cycle arrest and activated caspases-3, -8 and -9 besides PARP cleavage indicating onset of apoptosis and eventually leading to a significant reduction in tumor volume [139].

### 4.2. Vasohibins

**Figure 3:**
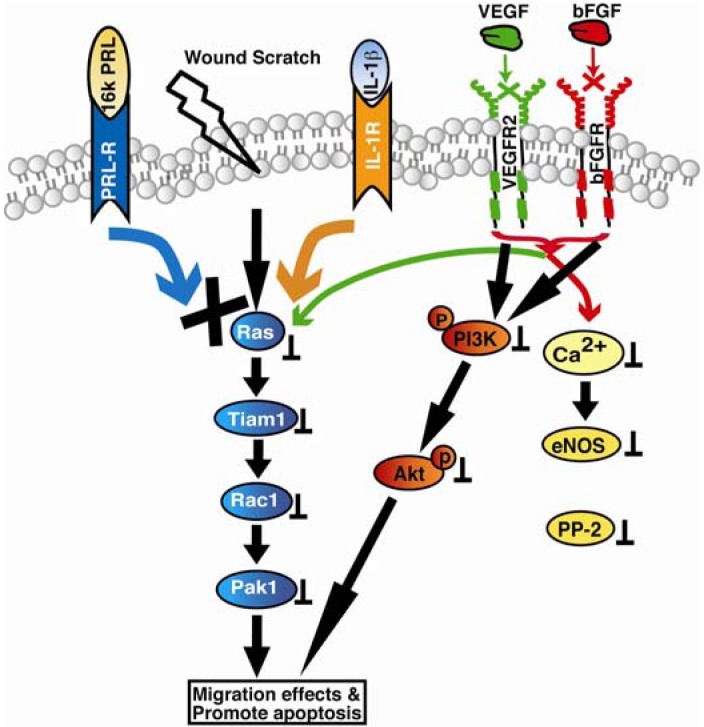
Illustration of vasoinhibin mediated angioinhibitory signaling.

Vasohibin-1 (VASH1) was identified as a VEGF induced angiogenesis inhibitor whose expression was observed in endothelial cells both under physiological and pathological conditions associated with angiogenesis, and also in other cell types. Vasohibin-1 was reported to inhibit endothelial cell proliferation, migration and tube formation *in vitro* and angiogenesis *in vivo* [140]. Although expression of vasohibin-1 was observed in endothelial cells during the embryo development stages, because it is induced by VEGF and FGF, its expression was observed exclusively in newly formed blood vessels where angiogenesis terminates [140-142]. Steady state expression of vasohibin-1 was also reported in adult bone marrow derived haematopoietic stem cells[143]. Expression of vasohibin-1 in endothelial cells was also observed in various solid tumors, atherosclerotic lesions, age-dependent macular degeneration, diabetic retinopathy, and rheumatoid arthritis [144-151]. However, the molecular mechanisms of vasohibins remained largely unclear. Both human and murine vasohibin-1 proteins showed increased apoptosis in mouse fibroblasts, however only murine isoform was found to inhibit migration of human endothelial cells through scratch assay [152]. Recombinant vasohibin-1 protein when applied exogenously or when its murine isoform was overexpressed intracellular, strong inhibition of angiogenic sprouting of HUVEC spheroids was observed in the three-dimensional collagen gel [152]. Moreover, vasohibin-1 was demonstrated to be strongly upregulated by VEGF in the mouse retinopathy model *in vivo*. The effects of Vasohibin-1 as reported using corneal micro pocket assay showed its distinct antiangiogenic and antilymphangiogenic activity [153]. In addition, they also found that vasohibin-1 inhibits tumor lymphangiogenesis and lymph node metastasis. Moreover, tumor angiogenesis was reported to be inhibited when LLC cells were transfected with vasohibin-1. It was interesting to note that vasohibin-1 did not affect tyrosine phosphorylation of KDR or activation of ERK1/2 when HUVECs were stimulated with VEGF. However, hypoxia and TNF-α inhibited VEGF-stimulated induction of vasohibin-1 in endothelial cells. Vasohibin-2 shares over 52% similarity with vasohibin-1 and is expressed preferentially in mononuclear cells. In contrast to vasohibin-1, vasohibin-2 null mice showed increased angiogenesis [142]. The genetic organization of vasohibin-2 gene in the parasite *Schistosoma mansoni*, revealed identification of 14 different alternatively spliced variants that encode seven different protein isoforms [154]. Signaling mechanisms of angioinhibitor vasoinhibin are shown in Figure 3.

## 5. Conclusions

A new branch of cancer research emerged over forty years ago in the early 70s with the hypothesis of Judah Folkman from Harvard Medical School and with the discovery of thrombospondins. Since then, many different endogenous angioinhibitors have been discovered, with over 27 of them that have been identified for having antiangiogenic functions. The endogenous angioinhibitors described in the present review have been well characterized for their role in antiangiogenic functions. These molecules inhibit endothelial angiogenic functions that include cell migration, proliferation, and tube formation *in vitro*, besides inhibiting matrigel plug angiogenesis, CAM or tumor angiogenesis *in vivo*. These molecules typically mediate their antiangiogenic signaling by binding to cell surface integrins on endothelial cells and affect expression of kinases involved in cell survival pathways that include MAPK and PI3K, *etc.*, which are induced by growth factors, especially VEGF. Besides they also affect expression of proangiogenic molecules such as MMPs, COX2 eNOS, *etc.*, eventually regulating the cellular physiological processes and inhibiting cell growth. Also, some of these endogenous angioinhibitors were reported to induce apoptotic pathways in endothelial cells by upregulating caspases and the associated downstream signaling. As described before, some of these proteins not only exert their functions by interacting with integrins on cell surface, but they also get internalized and affect proangiogenic pathways. Also many of these proteins were reported to have direct effects on tumor cells as well, indicating the many diverse mechanisms of actions of these endogenous molecules in inhibiting the growth of new blood vessels. Although the mechanisms of action and the pharmacological and pharmacodynamic studies on these endogenous antiangiogenic molecules are yet to be carried out to fully understand their antiangiogenic and antitumor properties before being tested through clinical trials, in light of their multiple mechanisms of actions, these molecules are gaining the significance of having high therapeutic potential with their promising role in combination therapy for cancer treatment. The summary of the above matrix derived and plasma derived angioinhibitors and their receptors with possible mechanism of actions are stated in Table 1.

## 6. Future Perspectives

The very existence of these endogenous angioinhibitors in the normal healthy individuals indicates a new line of host defense system to maintain angiogenic balance, and this property could be potentially applied to prevent progression of angiogenesis related diseases. The striking feature that makes these endogenous angioinhibitors effective for use in cancer therapy is because of their little or no side effects and drug resistance. Several gene therapy options are available to apply antiangiogenic therapy that can be targeted to the disease locus. Since tumor recurrence is common to chemotherapeutic regimes, treating patients with these endogenous antiangiogenic agents in combination with chemotherapy or immunotherapy could prove to be an efficient means to prevent tumor recurrence. Use of these endogenous antiangiogenic agents in combination with chemotherapy is emerging as an effective option to address several angiogenesis related diseases. With about 27 of these endogenous antiangiogenic agents identified for their potential role in cancer treatment, and with many of them are presently under preclinical and clinical phase trials, the applicability of these endogenous molecules for the treatment of tumor angiogenesis appears very promising.

## Figures and Tables

***Tumstatin***: It binds to αVβ3 and α3β1 integrins and inhibits FAK, Akt, PI3-K, mTOR, eIF4E and 4E-BP1 phosphorylation to decrease endothelial cell protein synthesis and proliferation. In addition tumstatin also inhibits transcription factor-NFκB mediated signaling in hypoxic conditions in endothelial cells leading to the inhibition of COX-2, bFGF and VEGF expressions, resulting in inhibition of tumor angiogenesis and LASER induced CNV in in-vivo mice models. ***Arresten***: It binds to α1β1 integrin and inhibits FAK, Ras, Raf, ERK1 and p38-MAPK phosphorylation that leads to inhibition of HIF-1α and VEGF expression resulting in inhibition of endothelial cell proliferation, migration and tube formation. In addition arresten also initiates activation of caspase-9 and -8, leading to activation of caspase-3, PARP cleavage in two different ways: (i) arresten activates caspase-3 and caspase-9 directly through inhibition of FAK/p38-MAPK/Bcl-2/Bcl-x_L_ in proliferating endothelial cells; (ii) possible, α1β1 integrin cross talk with Fas-Ligand through mitochondrial pathway leads to activation of caspase-8 and-3 in proliferating endothelial cells. ***Endostatin***: It binds to α5β1 integrin and inhibits FAK phosphorylation that leads to of Ras, Raf, ERK1 and p38-MAPK phosphorylation inhibition that leads to inhibition of endothelial cell migration and promotes apoptosis in LASER induced CNV in *in vivo* mice models. ***Canstatin***: It binds to integrins αVβ5/αVβ3 and inhibits two different apoptotic pathways, involving activation of caspase-9 and caspase-8 that leads to activation of caspase-3. Canstatin activates procaspase-9 not only through inhibition of the FAK/PI3K/Akt pathways but also by integrins cross talking mitochondrial pathway through Fas-Ligand dependent caspase-8 activation leads to endothelial cell apoptosis in LASER induced CNV models. ***Endorepellin***: It binds to α2β1 integrins and VEGFR-2, activates SHP-1 mediated cAMP-PKA/FAK/p38-MAPK/Hsp27 signaling pathway.

***Angiostatin***: It binds to αVβ3 integrin and inhibit FAK phosphorylation and ATP synthase in endothelial cells. ***Thrombospondins (TSPs)***: Bind to CD36 and integrin associated protein (IAP) promoting Src-family protein kinases/Caspase-3/p38-MAPK signaling, leading to activation of apoptosis In addition TSPs also binds to heparan sulfate proteoglycans (HSPGs), CD47, α3β1 and promotes TGF-β activation that leading to tumor cell death.

***Vasoinhibin (16Kda PRL)***: It binds prolactin receptor (PRL-R) and interleukin-1 receptor (IL-1β) and promotes Ras/Tiam-1/Rac-1/Pak1 and Bcl-_XL_ mediated apoptosis. In addition vasoinhibins also inhibit VEGF and bFGF mediated PI3K/Akt, and Ca2+/eNOS/protein phosphatase 2 signaling.

**Table 1:** Extracellular Matrix derived Endogenous angioinhibitors and their mode of action.

**Angioinhibitor**	**Parent molecule**	**Receptors**	**Mode of action**
Angiostatin	Plasminogen	ATP synthases, αVβ3 integrin, angiomotin	αVβ3 integrin mediated apoptosis in endothelial cells, ATP synthase α1β1 integrin dependent endothelial
Arresten	Type IV Collagen α1 NC1 domain	α1β1 integrin, HSPG	Raf/MEK/ERK1/2/p38-MAPK, HIF-1 inhibition and integrin independent MMP-2 activation inhibition
Canstatin	Type IV Collagen α2 NC1 domain	αVβ3, αVβ5 integrins and cross talk with, Fas Ligand	Integrins dependent inhibition of Akt/FAK/mToR, eIF-4EBP-1 activation, inhibition of caspase-8 and -9 activation and Ribosomal S6-kinase
Endorepellin	Perlecan	α2β1 integrins, lipid rafts, caveolin	Inhibition of cAMP-PKA/FAK/p38-MAPK/Hsp27, SHP-1, Ca2+ signaling
Endostatin	Type XVIII Collagen NC1 domain	αVβ1/α5β1 integrins, HSP, glypican, caveolin-1	Inhibition of Ras/Raf/KDR/Flk-1/ERK/p38-MAPK/p125 FAK/HIF-1α/Ephrin/TNFα/ NF-κβ, Wnt signaling
PEX domain	MMP-2	αVβ3 integrin	Interacts with αVβ3 integrins and prevents MMP-2 binding to αVβ3 integrins
Prothrombin Kringle-2	Prothrombin	αVβ3 integrin	Inhibits VEGF, MMP-2 and 9 expression, affects EC growth.
Thrombospondins	TSP	α3β1, CD47, HSPG, CD36, IAP	Inhibition of Src-family kinases/ Caspase-3/p38 MAPK, TGF-β signaling
Tumstatin	Type IV Collagen α3 NC1 domain	αVβ3, α3β1, α6β1 integrins, CD47/IAP	Inhibition of FAK/Akt/PI3K/mTOR/eIF-4EBP1 signaling; NFκB, COX-2 dependent tumor angiogenesis inhibition signaling
Vasoinhibins	Prolactin, growth hormone	Not known	Sos/Ras/MAPK or eNOS/Raf/MAPK, Ca2+/eNOS/protein phosphatase 2, Ras/ Tiam-1/Rac1/Pak1, Bcl-XL, NF-κβ, caspases

Akt: protein kinase B, Bcl-XL: B-cell lymphoma-extra large, bFGF: basic fibroblast growth factor, CD47 Integrin Associated Protein, COX-2: cyclooxygenase-2, eIF-4EBP-1: eukaryotic translation initiation factor-4E binding protein-1, eNOS: endothelial nitric oxide synthase, ECs: endothelial cells, ERK1/2: extracellular signal-regulated kinase1/2, FAK: focal adhesion kinase, HIF-1a: hypoxia inducible factor-1a, Hsp: heat shock protein, HSPG: Heparan sulfate proteoglycan, IAP: integrin associated protein, IKK: IκB kinase, KDR: kinase insert domain receptor, MAPK: Mitogen activated protein kinase, MEK: MAPK-ERK kinase, MIP: macrophage inflammatory protein-1/-2, MMPs: matrix metallo proteinases, mTOR: mammalian target of rapamycin, NF-κβ: nuclear factor kappa β, PEX: noncatalytic carboxy-terminal hemopexin-like domain of MMP, PI3K: phosphatidyl inositol 3-kinase, Rac: Ras-related C3 botulinin toxin susbtrate 1, Raf: Ras activated factor, Ras: Rat sarcoma, SHP: Src homology region 2 domain-containing phopshatase, Src: Schmidt-Ruppin A-2 sarcoma viral oncogene homolog, TIAM: T-lymphoma invasion and metastasis-inducing protein, TGF-β: transforming growth factor β, TNFα: tumor necrosis factor α, TSP: thrombospondin.
